# Analytical Validation of an Automated Point‐of‐Care Immunoassay for the Measurement of Canine Pancreatic Lipase Immunoreactivity Concentration (Vcheck cPL 2.0)

**DOI:** 10.1111/vcp.70106

**Published:** 2026-05-01

**Authors:** Isabel Mendoza‐White, Joerg M. Steiner, Harry Cridge

**Affiliations:** ^1^ Department of Small Animal Clinical Sciences, College of Veterinary Medicine Michigan State University East Lansing Michigan USA; ^2^ Gastrointestinal Laboratory, Department of Small Animal Clinical Sciences, College of Veterinary Medicine and Biomedical Sciences Texas A&M University College Station Texas USA

**Keywords:** dog, immunoassay, pancreatitis, PLI, point‐of‐care

## Abstract

**Background:**

Pancreatic lipase assays are commonly utilized in the diagnostic approach to suspected pancreatitis in dogs. While a previously available point‐of‐care assay for the quantification of canine pancreatic lipase immunoreactivity (Vcheck cPL 1.0) performed sub‐optimally, the updated version (Vcheck cPL 2.0) lacks independent validation.

**Objective:**

To evaluate the analytic validity of the Vcheck cPL 2.0 assay.

**Methods:**

Linearity was assessed via dilutional parallelism. Intra‐ and inter‐assay variability were assessed by calculating coefficients of variation across replicates on the same day and across consecutive days, respectively. Interference studies were performed to assess the effects of bilirubin, hemoglobin, and lipid presence in test samples on assay performance.

**Results:**

Linearity appeared within target parameters at pancreatic lipase concentrations up to ~800 md/dL (mean O/E ratio: 0.968 ± 0.104) but was unacceptable at pancreatic lipase concentrations > 2000 mg/dL (mean O/E ratio: 1.432 ± 0.178). Intra‐assay precision (median coefficient of variation 7.2%) was acceptable; however, inter‐assay precision was sub‐optimal (coefficient of variation > 10%) in 50% of samples analyzed. Variable precision may result in different diagnostic interpretations. Icterus and lipemia did not impact assay results. However, hemolysis, when severe (375–500 mg/dL), may impact test results (*p* < 0.005).

**Conclusions:**

The Vcheck cPL 2.0 shows improved precision compared to previous versions. Linearity was unacceptable at higher pancreatic lipase concentrations and hemolysis may impact Vcheck cPL 2.0 assay results.

## 1 Introduction

Pancreatitis is the most common disorder of the exocrine pancreas in dogs. While histopathology has traditionally been utilized to define the disease, it is associated with inherent limitations, and clinicians are therefore often reliant on integration of other case data to diagnose pancreatitis, including the quantification of pancreatic lipase [1]. In health < 1% of pancreatic lipase enters the systemic circulation; however, during pancreatic inflammation large amounts of pancreatic lipase leak into the systemic circulation, making it a good biomarker of pancreatic injury & pancreatitis [1, 2].

Pancreatic lipase concentrations can be quantified via immunologic assays that use species‐specific antibodies directed against pancreatic lipase, or lipase activity can be quantified via catalytic assays [3]. The biochemical reference standard for pancreatic lipase quantification is considered the canine pancreas‐specific lipase immunoreactivity (Spec cPL) assay. This assay is available via reference laboratories and, as such, results may take time to return. This has prompted the development of point‐of‐care assays that allow for time‐efficient quantification of pancreatic lipase activity or concentration. One such assay is the SNAP cPL, which is a semi‐quantitative point‐of‐care assay designed to be highly sensitive and suitable for ruling out pancreatitis, with positive results being followed by a quantitative pancreatic lipase assay [4]. The Vcheck cPL (Bionote, Hwaseong‐si, Gyeonggi‐do, Republic of Korea) is an immunoassay designed for the point‐of‐care quantification of canine pancreatic lipase immunoreactivity. Its clinical performance has been reported to be equivalent to that of the SNAP cPL and Spec cPL assay [5, 6]. The manufacturer reports good correlation with the Spec cPL assay (*r* = 0.967, *p* < 0.00001) and a mean intra‐assay % coefficient of variation (%CV) across 3 replicates of 7.9% (range: 0.42%–18.8%) based on a university cohort [7]. However analytical validation of the original (“1.0”) assay revealed that it had suboptimal linearity and precision, prompting questions regarding the routine use of the assay [8, 9]. Since that time the manufacturer has released a “2.0” version of the assay. A recent study noted intra‐assay %CV of 12.1% and inter‐assay %CV of 6.4% [10]. Unfortunately, it is unclear whether those authors utilized the Vcheck cPL 1.0 or 2.0 assay in their investigation. Thus, the aims of our study were to evaluate the analytical performance of the Vcheck cPL 2.0 assay for the measurement of pancreatic lipase in canine serum. We hypothesized that the Vcheck cPL 2.0 assay would have improved analytical performance relative to the 1.0 assay.

## 2 Materials and Methods

### 2.1 Samples

Thirteen residual serum samples were used for this study. Samples originated from the diagnostic caseload for quantification of Spec cPL at the Texas A&M University Gastrointestinal Laboratory and represented a range of Spec cPL concentrations (44–1951 μg/L). All samples were confirmed to be free of visible hemolysis, lipemia, and icterus. Samples underwent a single freeze‐thaw at room temperature prior to analysis. Samples were stored at −80°C for < 6 months prior to analysis. An IACUC exemption was approved for this study due to the use of residual serum samples.

### 2.2 Vcheck cPL 2.0 Assay

The Vcheck cPL 2.0 assay was performed by a single investigator (IMW) in a laboratory setting and according to manufacturer instructions. Quality control cartridges were performed at the start of the study and when otherwise recommended by the analyzer as per manufacturer protocol. When Vcheck cPL 2.0 assay results were < 50 μg/L, the result was recorded as 49.9 μg/L for calculation purposes. The assay reports pancreatic lipase concentrations as ng/mL; however, throughout this manuscript, we use μg/L for consistency with the Spec cPL assay and to minimize potential confusion.

### 2.3 Linearity

Linearity was assessed via dilutional parallelism and saline dilution. Five surplus serum samples were utilized, two of which had serum Spec cPL concentrations > 400 μg/L (considered consistent with a diagnosis of pancreatitis), one had a serum Spec cPL concentration in the diagnostic gray zone (200–400 μg/L), and two of which had normal Spec cPL concentrations (< 200 μg/L). Dilutional linearity was assessed by calculating the observed to expected (O/E) ratio and was compared to a target standard of 0.8–1.2, as recommended and utilized in a recent study [11]. Dilutional O/E ratios outside of these parameters (i.e., < 0.80 or > 1.20) were defined as unacceptable.

### 2.4 Precision and Total Error

The intra‐assay variability was assessed using 8 replicates of 6 samples on the same day. Samples were thawed at room temperature prior to quantification. The corresponding Spec cPL concentrations for these samples spanned the dynamic range of the reference assay from 149 to 1408 μg/L. Inter‐assay variability was assessed using 5 replicates of 6 samples on consecutive days, using frozen aliquots. The corresponding Spec cPL concentrations for these samples spanned the dynamic range of the reference assay from 44 to 1734 μg/L. The mean, standard deviation (SD), and coefficients of variation (CV) were calculated and compared with the target %CVs utilized in a recent study [11]. Namely, a target CV was defined as < 10%, a sub‐optimal but acceptable CV was defined as 10% < %CV ≤ 20%, and a CV > 20% was deemed unacceptable [11]. %CV was calculated as (standard deviation/mean) × 100, based on non‐truncated calculated values for mean and standard deviation (SD). Bias was calculated as bias (%) = ([reference Spec cPL—mean measured Vcheck cPL concentration/reference Spec cPL concentration]) × 100. Total error (TE) was calculated as 2 × %CV + %Bias. As multiple samples were used to calculate precision, a total error was calculated and reported for each sample.

### 2.5 Interference Study

The effect of common interfering substances on Vcheck cPL 2.0 assay results was evaluated, including the effect of bilirubin (icterus), hemoglobin (hemolysis), and lipid (lipemia). A linear mixed‐effects model was used to assess if each of the interfering substances influenced Vcheck cPL 2.0 concentrations, using Spec cPL concentration as the response variable, interfering substance dilution as a fixed effect, and the sample as a random effect. Log transformation was applied to Vcheck cPL 2.0 results to satisfy the model assumptions. Model assumptions were assessed using plots of conditional model residuals. Statistical analysis was performed using R (version 4.5.0) with the package lme4 software [12, 13]. Significance for all statistical comparisons was set at *p <* 0.05.

Bilirubin (Scripps Laboratory, San Diego, CA, USA) was added at increasing concentrations (7.5, 15, and 22.5 mg/dL) to three serum samples (Vcheck cPL 2.0: 82.7, 503.3, and 926.2 μg/L). Each sample was also tested in the absence of the interfering substance (neat).

Hemoglobin (hemolyzed red blood cells from canine blood) was added at increasing concentrations (125, 250, 375, 375, and 500 mg/dL) to three serum samples (Vcheck cPL 2.0: 79.3, 520.7, and 761.0 μg/L). Each sample was also tested in the absence of an interfering substance (neat).

Lipid (Intralipid 30% IV emulsion, VWR, Radnor, PA, USA) was added at increasing concentrations (300 mg/dL, 600 mg/dL, 900 mg/dL, and 1200 mg/dL) to three serum samples (Vcheck cPL 2.0: 82.7, 581.7, and 1157.2 μg/L). Each sample was also tested in the absence of an interfering substance (neat).

### 2.6 Correlation Study

The correlation between reported Spec cPL concentration and the 1st Vcheck cPL 2.0 assay concentrations from the linearity and intra‐assay precision studies was calculated using Pearson correlation. Significance was set at *p <* 0.05.

## 3 Results

### 3.1 Linearity

The dilutional linearity study revealed O/E ratios of 0.785–1.683 with a mean ± SD of 1.104 ± 0.251 (Table 1). Dilutional linearity appeared appropriate within samples with undiluted pancreatic lipase concentrations beginning at up to ~800 mg/dL (mean O/E ratio: 0.968 ± 0.104) but was unacceptable at samples with undiluted pancreatic lipase concentrations beginning at > 2000 mg/dL (mean O/E ratio: 1.432 ± 0.178). At these high pancreatic lipase concentrations, all dilutions within the measurable range of the assay had O/E's outside of the targeted 0.80–1.20 range (Table 1).

**TABLE 1:** Linearity (Vcheck cPL 2.0).

Dilution	Observed (μg/L)	Expected (μg/L)	O/E ratio (%)
*Sample 1*			
Neat	159.9	—	—
1:2	78.9	80.0	98.6
1:4	49.9^a^ (< 50)	49.9^a^ (< 50)	N/A^a^
1:8	49.9^a^ (< 50)	49.9^a^ (< 50)	N/A^a^
*Sample 2*			
Neat	249.7	—	—
1:2	125.5	124.9	100.5
1:4	62.2	62.4	99.7
1:8	49.9^a^ (< 50)	49.9^a^ (< 50)	N/A^a^
*Sample 3*			
Neat	484.9	—	—
1:2	190.3	242.5	78.5
1:4	96.4	121.2	79.5
1:8	58.5	60.6	96.5
*Sample 4*			
Neat	741.2	—	—
1:2	351.4	370.6	94.8
1:4	159.4	185.3	86.0
1:8	98.7	92.7	106.5
*Sample 5*			
Neat	817.5	—	—
1:2	433.8	408.8	106.1
1:4	212.9	204.4	104.2
1:8	112.7	102.2	110.3
*Sample 6*			
Neat	> 2000	—	—
1:2	> 2000	—	—
1:4	1922.6	—	—
1:8	1204.9	961.3	125.3
1:16	731.9	480.7	152.3
*Sample 7*			
Neat	> 2000	—	—
1:2	1441.7	—	—
1:4	1023.1	720.9	141.9
1:8	606.6	360.5	168.3
1:16	230.9	180.3	128.1

^a^ O/E at 100% as below the limit of detection of assay, potentially over‐estimating assay performance.

### 3.2 Precision

For intra‐assay variability, sample 1 produced 7 replicates, reported cPLI as < 50 μg/L, which was converted to 49.9 μg/L for statistical analysis. The lowest reportable value for the VCheck cPL 2.0 assay (< 50 μg/L) is higher than that of the Spec cPL (< 30 μg/L). The median % CV was 7.6% for intra‐assay variability and 10.4% for inter‐assay variability. The highest intra‐assay %CV was reported in the equivocal Spec cPL result (Vcheck cPL 2.0; 379.3 μg/L; 28.3%), which resulted in 5/8 replicates being diagnostic for pancreatitis (> 400 μg/L), 2/8 replicates in the diagnostic gray zone (200–400 μg/L) and 1/8 replicates not consistent with a diagnosis of pancreatitis (< 200 μg/L). One of 6 (16.7%) samples had an intra‐assay %CV above the target of 10% (Table 2). The highest inter‐assay %CV was reported for the sample with the highest Spec cPL concentration (Vcheck cPL 2.0; 1334.7 μg/L; 14.6%). Three of 6 (50%) samples had an inter‐assay %CV above the target of 10% (Table 3).

**TABLE 2:** Intra‐assay %CV (Vcheck cPL 2.0).

Sample	Mean cPLI (μg/L)	SD	%CV
1	50.6	1.87	3.7
2	131.1	10.5	8.0
3	695.3	49.9	7.2
4	379.3	107.4	28.3
5	880.7	72.2	8.2
6	1291.7	90.3	7.0
Median %CV			7.6

**TABLE 3:** Inter‐assay %CV (Vcheck cPL 2.0).

Sample	Mean cPLI (ng/mL)	SD	%CV
1	51.8	3.7	7.2
2	117.3	15.5	13.2
3	698.6	75.2	10.8
4	413.6	31.6	7.6
5	850.5	85.1	10.0
6	1334.7	194.4	14.6
Median %CV			10.4

### 3.3 Effects of Interfering Substances

Lipemia (*p* = 0.129) and icterus (*p* = 0.071) did not result in significant changes in measured Vcheck cPL 2.0 assay results. In contrast, hemolysis (*p* < 0.001) may interfere with measured Vcheck cPL 2.0 assay results. When evaluating the degree of hemolysis, the addition of 125 mg/dL and 250 mg/dL hemoglobin did not result in changes in pancreatic lipase quantification (*p* = 0.432 and *p* = 0.124, respectively). In contrast, more significant hemolysis (375 mg/dL and 500 mg/dL) resulted in changes in measured Vcheck cPL 2.0 concentration (*p* = 0.003 and *p* = 0.001, respectively; Figure 1).

**FIGURE 1:**
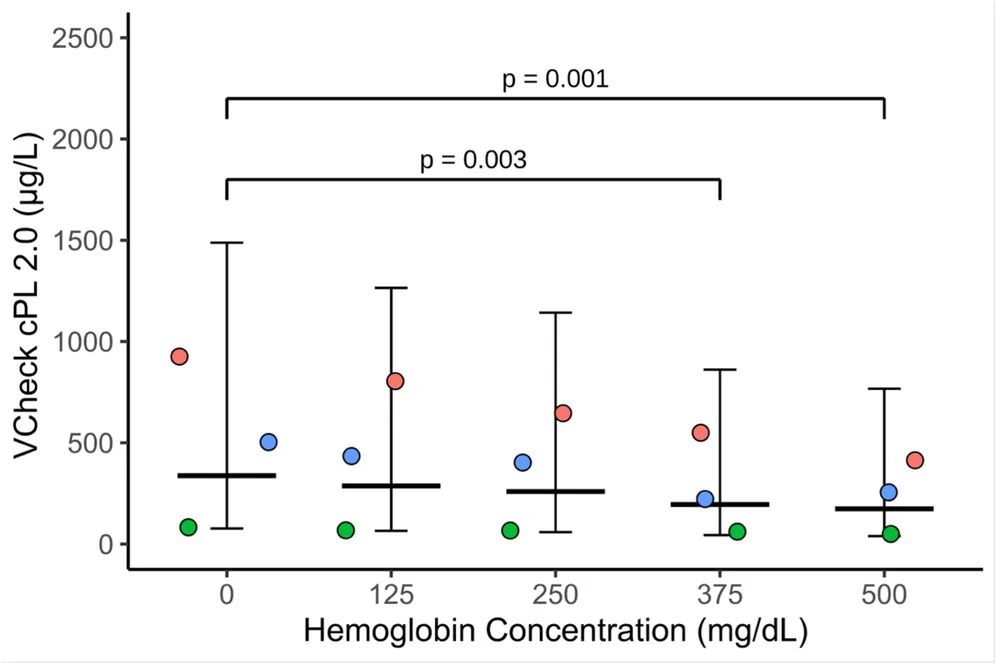
Influence of Hemoglobin on VCheck cPL 2.0 Assay. Hemolysis may interfere with measured Vcheck cPL 2.0 assay results. Measured pancreatic lipase concentrations were significantly lower at 375 mg/dL hemoglobin (*p* = 0.003) and 500 mg/dL (*p* = 0.001) when compared to quantification of pancreatic lipase in a neat sample. The thick horizontal bars represent model estimate means and after anti‐log‐back transformation and the thin horizontal bars represent model calculated error. Each colored dot represents a different sample.

### 3.4 Correlation

Vcheck cPL 2.0 assay results were positively correlated with Spec cPL assay concentrations (Pearson *r* = 0.9593, 95% CI: 0.8468–0.9897, *p* < 0.001); however, results appear more discrepant at increasing Spec cPL concentrations (Figure 2). Samples above the reportable range of each assay were excluded from comparison.

**FIGURE 2:**
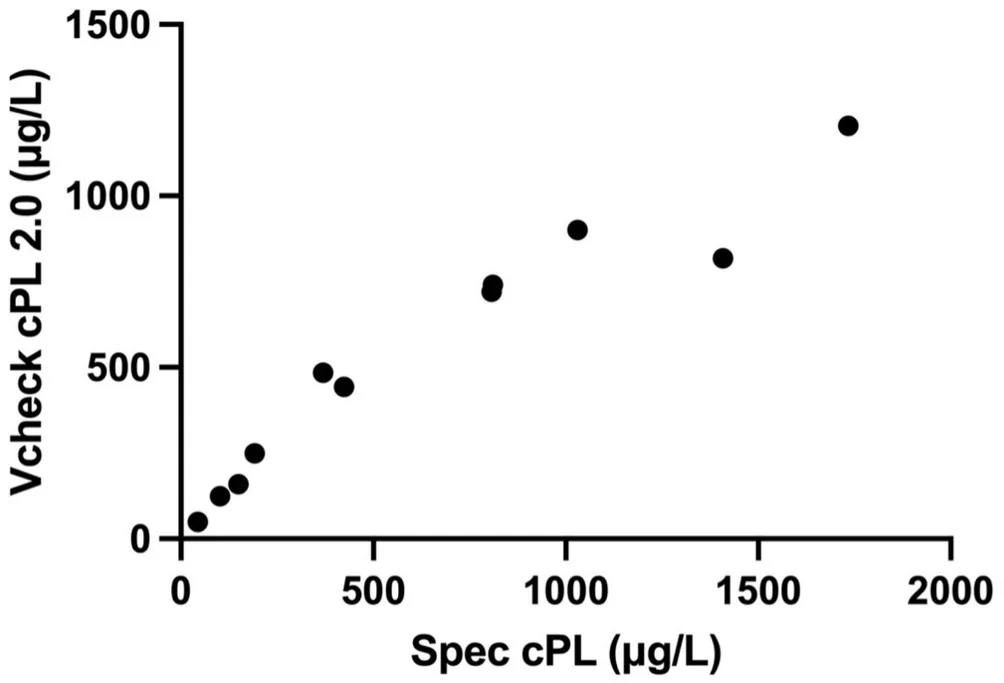
Correlation Between Vcheck cPL 2.0 and Spec cPL Assay Results. Vcheck cPL 2.0 assay results were positively correlated with Spec cPL assay concentrations (Pearson *r* = 0.9593, 95% CI: 0.8468–0.9897, *p* < 0.001); however, results appear more discrepant at increasing Spec cPL concentrations. Samples above the reportable range of each assay were excluded.

## 4 Discussion

The Vcheck cPL 2.0 assay used in this study had within‐target dilutional linearity and intra‐assay precision. Mean inter‐assay precision was suboptimal but acceptable, and hemolysis (375–500 mg/dL) was shown to falsely lower Vcheck cPL 2.0 concentrations.

The Vcheck cPL 2.0 assay had a median intra‐assay precision within target parameters (median %CV: 7.6). The intra‐assay precision reported here is similar to that reported in a non‐peer‐reviewed abstract (7.9%, range: 0.43%–18.18%) [7]. However, it is important to note that intra‐assay precision was sub‐optimal in a sample with an equivocal cPLI concentration, at 28.3%. While the exact reason for the marked difference in %CV for this sample relative to other samples is unknown, this is a clinically consequential finding and warrants additional investigation. Indeed, suboptimal precision at equivocal Spec cPL concentrations could result in diagnostic error in a clinical setting.

Inter‐assay precision (median %CV: 10.4) was less optimal (i.e., higher) than intra‐assay precision (median % CV: 7.6), and 50% of samples had inter‐assay precisions above previously published targets [11]. Inter‐assay precision was not reported by the manufacturer's validation study [7].

The impact of potential interfering substances on assay results is of critical importance in dogs with pancreatitis due to the high frequency of lipemic and hemolyzed samples in this patient population. Lipemia and icterus were not shown to impact measured Vcheck cPL 2.0 concentrations. The effects of lipemia on assay results were modeled using intra‐lipid, rather than naturally occurring lipemic samples, and this should be considered when interpreting study results. Unfortunately, marked hemolysis (375–500 mg/dL) was shown to impact measured Vcheck cPL 2.0 assay results, which may impact clinical use of the assay.

Linearity was assessed in this study using a saline dilution technique and single replicates. An alternate technique would be to use high and low pooled serum samples measured in duplicate or triplicate [14]. At VCheck cPL concentrations up to ~800 μg/L the O/Es were within the target of 0.8–1.2. However, when samples with higher pancreatic lipase concentrations were used to evaluate the dynamic range of the assay, the O/E's were above target parameters.

## 5 Conclusions

The Vcheck cPL 2.0 appears to have good linearity at low to moderate pancreatic lipase concentrations, but linearity is unacceptable at higher pancreatic lipase concentrations. Intra‐assay variability resulted in different diagnostic interpretations for an equivocal Spec cPL result sample. Significant hemolysis may result in false decreases in measured Vcheck cPL 2.0 assay results, and this should be considered if the assay is utilized in clinical practice.

## Conflicts of Interest

Dr. Steiner is the director of the Texas A&M Gastrointestinal Laboratory, which offers pancreatic lipase testing via the Spec cPL assay on a fee‐for‐service basis. Drs. Steiner and Cridge are paid speakers for IDEXX Laboratories. Dr. Steiner serves as a consultant for IDEXX Laboratories.
